# Nature-Inspired Structures Applied in Heat Transfer Enhancement and Drag Reduction

**DOI:** 10.3390/mi12060656

**Published:** 2021-06-03

**Authors:** Zhangyu Zhu, Juan Li, Hao Peng, Dongren Liu

**Affiliations:** 1 School of Mechanical and Electrical Engineering, Nanjing Forestry University, 159 Long Pan Road, Nanjing 210037, China; 15295566598@163.com; 2 School of Mechanical and Power Engineering, Nanjing Tech University, 30 South Pu Zhu Road, Nanjing 211816, China; phsight1@hotmail.com; 3 Mechanical Engineering College, Yangzhou University, 88 South University Ave., Yangzhou 225009, China; Drliu@yzu.edu.cn

**Keywords:** biomimetic structure, heat transfer enhancement, drag reduction, optimal design, heat exchanger

## Abstract

Heat exchangers are general equipment for energy exchange in the industrial field. Enhancing the heat transfer of a heat exchanger with low pump energy consumption is beneficial to the maximum utilization of energy. The optimization design for enhanced heat transfer structure is an effective method to improve the heat transfer coefficient. Present research shows that the biomimetic structures applied in different equipment could enhance heat transfer and reduce flow resistance significantly. Firstly, six biomimetic structures including the fractal-tree-like structure, conical column structure, hybrid wetting structure, scale structure, concave-convex structure and superhydrophobic micro-nano structure were summarized in this paper. The biomimetic structure characteristics and heat transfer enhancement and drag reduction mechanisms were analyzed. Secondly, four processing methods including photolithography, nanoimprinting, femtosecond laser processing and 3D printing were introduced as the reference of biomimetic structure machining. Finally, according to the systemic summary of the research review, the prospect of biomimetic heat transfer structure optimization was proposed.

## 1. Introduction

With the rapid development of human society, fossil energy has been gradually exhausted. All countries in the world are promoting the innovation of energy science and technology in the industrial field to increase energy utilization efficiency [1,2,3]. A heat exchanger is a general device for energy exchange. In the primary energy consumption, about 80% of the primary energy have to be utilized by converting into heat. Hence, the heat transfer efficiency of a heat exchanger directly affects the energy utilization efficiency. The heat transfer can be improved through the application of the enhanced heat transfer structures [4,5]. The present enhanced heat transfer structures usually increase the heat transfer coefficient while causing a significant increase in flow resistance, especially in the microscale heat transfer field with limited volume and mass [6,7].

As an emerging interdisciplinary subject combining biology, materials science and engineering technology, the bionics provides a new thinking way for energy conservation and consumption reduction [8,9]. The basic steps of the bionics research are to analyze the influence factors in practical engineering, and then to search and learn from the external structures or functional behaviors of animals and plants with homologous mechanisms in nature. Thus, the biomimetic structures are simplified and manufactured through processing methods for engineering applications [10,11,12].

In recent years, the phenomena of heat transfer enhancement in nature and non-smooth surface structures of organisms have inspired many researchers. The experimental or numerical simulation methods were used to study the bio-inspired structure applied in heat exchangers, traffic tools, agricultural machinery, etc. [13,14]. Furthermore, micro-nano processing technologies have been developed to satisfy the machining precision of the biomimetic structures. Therefore, the research progress was summarized for six biomimetic structures applied in heat transfer enhancement and drag reduction, and four micro-nano machining technologies were introduced. The prospect of biomimetic heat transfer structure optimization was proposed, hoping to provide a design reference for enhanced heat transfer structures of heat exchangers, especially miniature heat exchangers.

## 2. Biomimetic Enhanced Heat Transfer Structures

The heat transfer is ubiquitous during the natural biological system construction and life activities process. A long-term nature evolution could provide beneficial guidance for bionic optimal design of heat transfer structure.

### 2.1. Fractal-Tree-Like Structure

The fractal theory [15] is commonly used to describe similar fractal geometric structures in nature, which is employed widely to research the fractal-tree-like structure inspired by tree trunk lines, leaf veins distribution and human blood vessel networks for the structural design of heat sinks [16,17].

**Figure 1:**
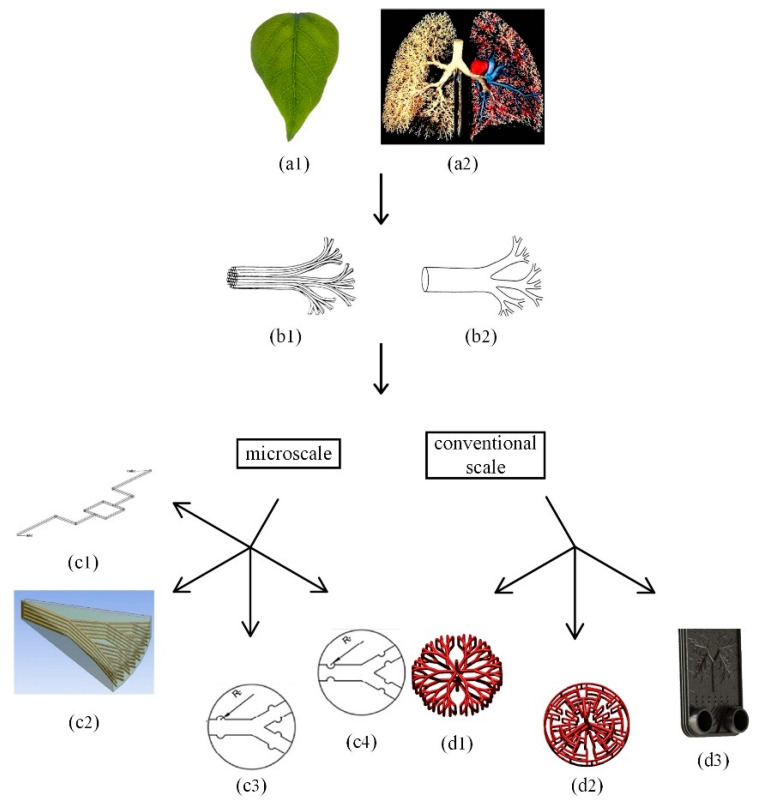
(**a1**) The image of leaf veins, (**a2**) The human lung model; (**b1**) The bifurcate structure of the vascular system in plants, (**b2**) The bifurcate structure of the vascular or tracheal system in animals; Fractal-like branching channel flow networks: (**c1**) The fractal-tree-like microchannel network [19], (**c2**) The overall shape of 5-layer fractal-tree-like microchannel [20], (**c3**) The tree-shaped microchannel heat sink with cavities [21], (**c4**) The tree-shaped microchannel heat sink with cavities [21]; (**d1**) The CAD diagram of Y-type heat exchanger [22], (**d2**) The CAD diagram of H-type heat exchanger [22], (**d3**) The plate heat exchanger with lung pattern [23].

Figure 1 and Table 1 show the structures of several fractal heat exchangers in recent years. The scale, shape and application tend to be diversified. Furthermore, the fractal-tree-like structure characteristics mainly include the branch number and angle [18], the channel aspect ratio [19], layer number [20] and shape [21,22,23], which can affect the fractal heat exchanger performance.

Through the comparison between the fractal-tree-like microchannel and the traditional parallel microchannel, the branch number and angle are worth discussing [18]. It proved that the heat transfer performance of the fractal-tree-like microchannel first increased and then decreased in the aspect ratio range of 0.3–1 [19]. The thermal resistance was effectively reduced when the single-layer fractal-tree-like silicon microchannel was turned into the multi-layer structure [20]. The fractal-tree-like microchannel with micro-rib contributed to destroy the flow boundary layer near the wall and increase the Nusselt number [21]. The total heat flux of the spiral-tube heat exchanger increased by 23% for H-type fractal-tree-like channel, comparing with the conventional spiral-tube [22]. The plate heat exchanger with lung structure had smaller volume and higher heat transfer coefficient than that of the corrugated plate-type heat exchanger [23].

### 2.2. Conical Column Structure

**Figure 2:**
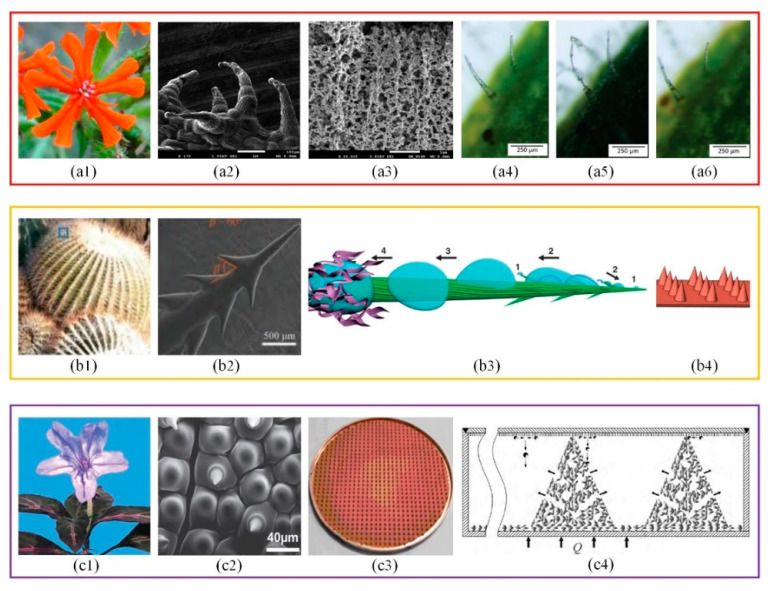
(**a1**) The image of Lychnis sibirica, (**a2**) The field emission SEM image of intact hairs [29], (**a3**) The sponge-like microfibrillar texture within the hair [29], (**a4**–**a6**) Hair deformation by water droplets [29]; (**b1**) The image of Cactus, (**b2**) SEM image of cactus spiny surface [32], (**b3**) The mechanism model of water droplet movement on cactus spiny surface [34], (**b4**) The image of superhydrophilic mastoids [38]; (**c1**) The image of Ruellia devosiana, (**c2**) The SEM image of Ruellia devosiana leaf [37], (**c3**) The image of the flat heat pipe with convex structure [39], (**c4**) Structure and working principle of the flat heat pipe with convex structure [39].

The condensation caused by vapor-liquid phase change is a common natural phenomenon [24,25]. There are two main condensation modes: dropwise condensation and film condensation. The condensate film is a thermal resistance carrier for the heat exchange between the steam and wall surfaces. The dropwise condensation has five to ten times higher heat transfer coefficient than that of the film condensation. This is because the condensate exists in the form of droplets, which can effectively break away from the wall and promote dropwise condensation. However, dropwise condensation is so unstable that it is difficult to maintain. The special structures on plant surfaces can remove condensate droplets effectively and continuously by means of absorption or self-jumping, which itself has aroused much attention of scholars [26,27,28], as shown in Figure 2.

The hollow hairs on the surface of Lychnis sibirica (Figure 2(a1)) appear to be conical column structures (Figure 2(a2,a3)). These conical column structures are utilized by Lychnis sibirica to absorb moisture from air, store moisture in the interior, and then bend hairs to release moisture to the plant in dry environments [29] (Figure 2(a4–a6)). The ability to survive in the desert of Cactus is closely related to its excellent vapor condensation and collection system [30,31,32]. The surface of Cactus (Figure 2(b1)) is uniformly covered with conical column structures (Figure 2(b2)). Each conical column contains barbs, grooves and trichomes [33,34]. During moisture collection, the uneven curvature of the conical column structure leads to the formation of the gradient of the Laplace pressure and drives droplets to slide spontaneously from the tip to root. Then, droplets are absorbed by trichomes through capillary action [35,36] (Figure 2(b3)). Similar conical column structures also appear on the surface of Ruellia devosiana [37] (Figure 2(c1,c2). It proved that the superhydrophobic surfaces with conical column structures (Figure 2(b4,c3,c4) showed more excellent condensing heat transfer performance [38,39].

**Figure 3:**
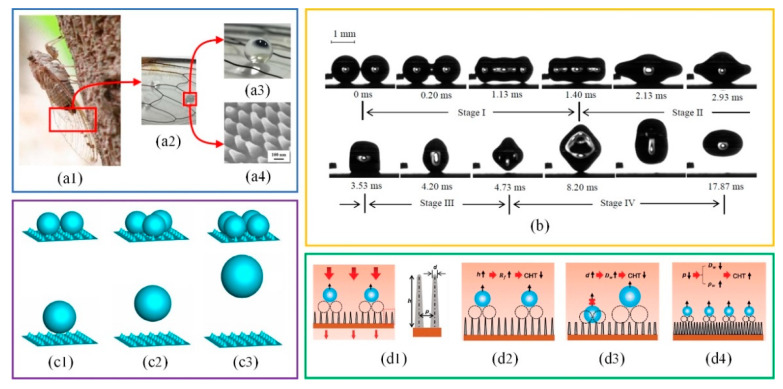
(**a1**) The image of cicada, (**a2**,**a3**) The wetting state of the local structure of cicada wings [41], (**a4**) The field emission SEM image of cicada wings [42]; (**b**) The coalescence-induced droplet jumping on superhydrophobic surfaces [43]; (**c1**–**c3**) The influence of the number of droplets on droplet jumping height [45]; (**d1**) A model of condensate self-propelling nanoneedle array structure with specific geometric parameters: interspace *p*, tip size *d* and height *h* [47], (**d2**–**d4**) The influence of *h*, *d* and *p* on the departure diameters *D_w_*, density *ρ_w_* of droplets and film-layer thermal resistance *R_f_* either of which is important to affect condensation heat transfer [47].

The phenomenon of droplets combining and jumping on a superhydrophobic surface provides a new research direction for dropwise condensation improvement. In order to fly in foggy weather, cicadas have to condense moisture in the air by evolving specific structural wings, as shown in Figure 3. The tiny condensate droplets combine together and jump off the surface of cicada wings which remove pollutants and keep wings dry [40,41] (Figure 3(a1–a3)). This self-cleaning behavior is done by the coordination of the conical column structures and hydrophobic wax layer on the surface of cicada wings [42] (Figure 3(a4)). The self-jumping of droplets generally includes the growth of the liquid bridge, the impact between the liquid bridge and wall, the contraction of the liquid bridge into droplets and the separation of droplets from the material surface [43] (Figure 3b). The critical radius of self-jumping droplets is positively correlated with the solid-liquid contact area and negatively correlated with the contact angle of the material surface [44]. Both the number and the distribution of combined droplets are important factors affecting the self-jumping speed [45,46] (Figure 3(c1–c3)). By adjusting the height, tip size and interspace of conical column structures (Figure 3(d1–d4)), Wang et al. [47] obtained 320% enhancement on the condensation heat transfer coefficient compared with the smooth hydrophobic surface.

### 2.3. Hybrid Wetting Structure

Wettability is one of important features for a solid surface, which can be divided into hydrophobic and hydrophilic [48,49,50]. The condensate droplets separate easily on the hydrophobic surface. However, the condensation amount is small due to the high nucleation barrier. In contrast, the condensate droplets are difficult to separate on the hydrophilic surface, and the condensation amount is large due to the low nucleation barrier [51,52,53]. Therefore, it is reasonable to distribute and regulate the wettability of structures for reducing condensate droplets nucleation barrier and increasing nucleation density.

**Figure 4:**
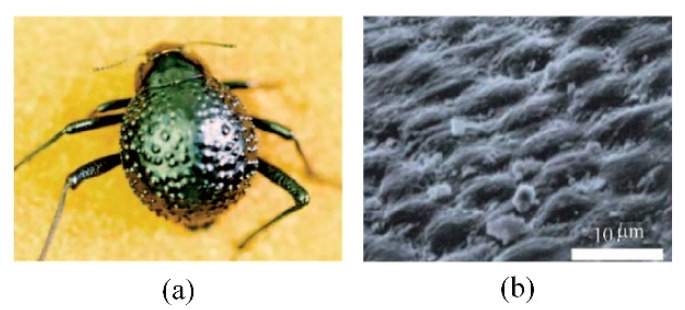
(**a**) The image of Namib desert beetle; (**b**) The SEM image of Namib desert beetle back [54].

The Namib desert beetle, which lives in an arid area, has a special hydrophilic and hydrophobic composite structure on its back, as shown in Figure 4. The hydrophilic protrusions can gather droplets suspended in the air and hydrophobic grooves can ensure that droplets flow to mouthparts after becoming large enough [54,55].

**Figure 5:**
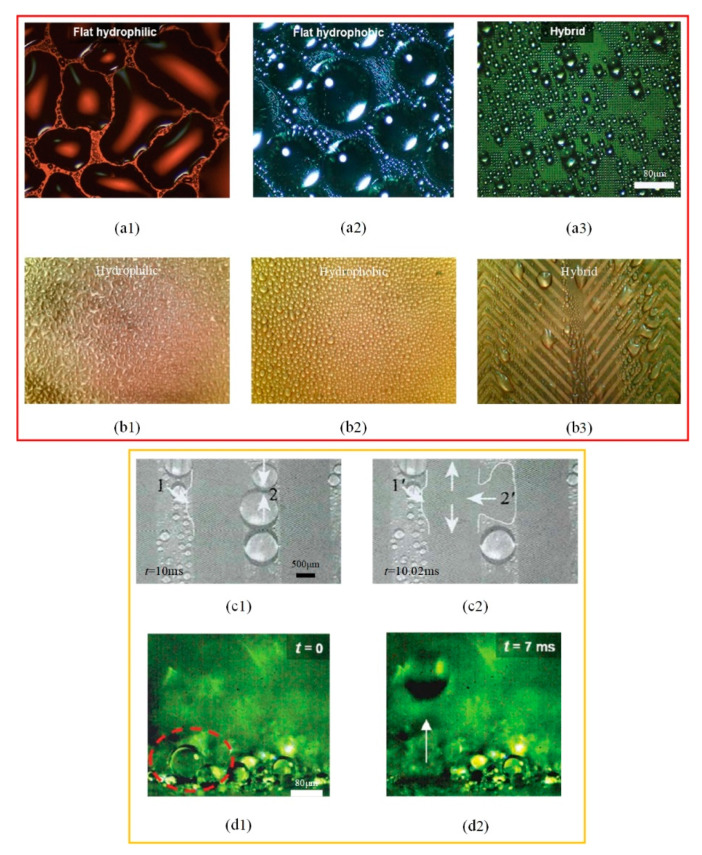
The condensate flow pattern on various surfaces with (**a1**,**b1**) hydrophilic [56,57]; (**a2**,**b2**) hydrophobic [56,57]; (**a3**,**b3**) different hybrid pattern [56,57]; (**c1**,**c2**) Coalescence droplets moving from superhydrophobic to superhydrophilic region [63]; (**d1**,**d2**) Snapshots showing spontaneous droplet jumping [56].

Referring to moisture collection of the Namib desert beetle, a variety of hydrophilic and hydrophobic composite structures were designed, as shown in Table 2. Compared with hydrophilic or hydrophobic surfaces, the heat transfer performance of the hybrid wetting surfaces is greatly enhanced [56,57,58,59]. It shows that there is no obvious film condensation on the hybrid wetting surface in Figure 5. In addition, the condensate droplets in the hydrophobic region are gathered in the adjacent hydrophilic region. Then, the hydrophobic surface continues to be exposed, so as to provide conditions for the dropwise condensation (Figure 5(a1–a3,b1–b3)). The condensate droplets on the hybrid wetting surface (Figure 5(b3)) and left surfaces (Figure 5(c1)) formed by the hydrophilic region flow along channels under the action of gravity (Figure 5(c1,c2)). Differing from that behavior, the condensate droplets on the hybrid wetting surface (Figure 5(a3)) could be removed through self-jumping supported by the excess surface energy after combining (Figure 5(d1,d2)). Furthermore, the pattern shape, inclined angle, spatial layout, fractional area and other structural parameters of the hydrophilic and hydrophobic region are important factors affecting the heat transfer performance of the hybrid wetting structures. Choo et al. [60] fabricated four kinds of superhydrophilic ZnO nanorod arrays on superhydrophobic TiO_2_ nanorods in the form of dot, mesh, line and branch, respectively. The hybrid wetting surface with dot patterns had the best condensation efficiency and the increase of surface inclined angle was beneficial. When the parallel-stripes patterns dipped to the width direction of the substrate with the inclined angle of sixty degrees, the hybrid wetting surface had the higher condensation heat transfer coefficient [61]. Mahapatra et al. [62] pointed out that both interdigitated and staggered line patterns showed better condensation heat transfer performance than the straight line patterns. The heat transfer enhancement rate of the staggered line patterns was higher than that of the interdigitated line patterns. Excessively increasing the area of the superhydrophilic region did not contribute to the condensation efficiency [62,63].

## 3. Biomimetic Flow Resistance Reduction Structures

In general, the flow resistance in the heat exchanger is mainly attributed to the internal friction caused by the viscosity of the fluid and the form drag caused by the shape of the fixed wall. The flow resistance determines the consumption of pump power, which is an important index to evaluate the overall performance of heat exchanger. Therefore, the design of enhanced heat transfer structure should not only pursue the improvement of heat transfer coefficient, but also strictly control the increase of flow resistance. There are some special structures on the organism surface in direct contact with the external fluid. Biomimetic technology improves fluid flow by simulating and designing non-smooth structures similar with the surface morphology of animals and plants.

### 3.1. Scale Structure

**Figure 6:**
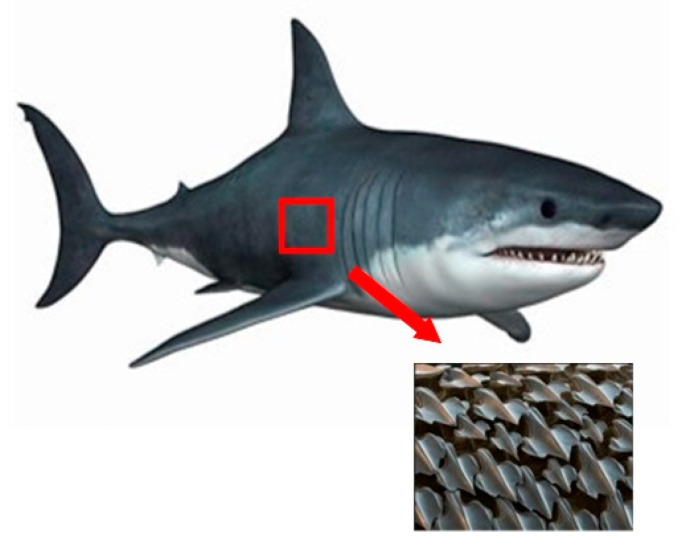
The image of a shark and its skin.

Fish is an important research object of bionics, and its excellent underwater locomotion ability has been widely noticed [64,65]. Sharks in the ocean are able to swim quickly mainly because of the non-smooth scale structures covering their skin, as shown in Figure 6 [66,67].

Inspired by shark skins, the grooves have been proposed, which could change the flow pattern in the turbulent boundary layer and reduce the viscous resistance of the fluid [68,69]. Grooves with triangular, trapezoidal, semicircular, rectangular, blade and sinusoidal sections have been designed [70,71,72,73]. The drag reduction rates of four textured surfaces with V-shaped, saw tooth, rectangular and semi-circular sections were examined. In these four geometries, the surface with saw tooth grooves had the best drag reduction efficiency [73]. The influences of the grooves parameters on the drag reduction rate have been discussed [74,75,76]. Martin et al. [74] established three surfaces with blade, sawtooth, scalloped grooves decorated along flow direction and vertical flow direction discontinuously. For all three grooves, the drag reduction rate increased first and then decreased with the increase of dimensionless spacing or height. For sinusoidal grooves, the variation of drag reduction rate with the dimensionless amplitude was similar to that of dimensionless structural parameters in Martin’s study. Smaller dimensionless wavelengths were not helpful for drag reduction. The use of polymer drag reducers or surfactants could also increase the drag reduction rate of the grooves [77,78,79]. The drag reduction rate of a biomimetic riblet surface increased by 6% after grafting the drag reduction agent polyacrylamide [77]. Table 3 shows several biomimetic shark-skin grooves and maximum drag reduction rates in recent years.

**Figure 7:**
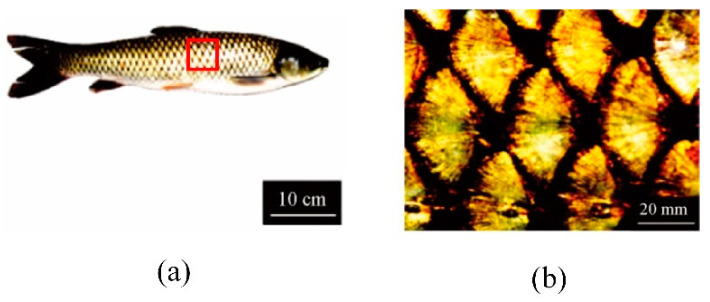
(**a**) The image of grass carp; (**b**) The macrostructure of overlapping scales [80].

In addition, the scale structures of fish living in different environments are different from each other. Grass carp is a common freshwater fish with multilevel structure scales, as shown in Figure 7 [80]. The grass carp body is covered by periodic scales and the mutual coverage of the scales is about 2/3 (Figure 7b). The exposed parts of scales are fan-shaped. The microscopic morphology of the exposed parts of scales shows that some “crescent-like” ridge distribution in an orderly manner. Based on the structure, Dey et al. [81] applied the fan-shaped scale in the microchannel. It indicated that the friction coefficient was reduced by up to 5% compared with that of the smooth microchannel. Wu et al. [82] established a 3-D biomimetic surface model with simplified crescent-like ridge and obtained a drag reduction rate of 3.014% through dynamic finite element analysis. This drag reduction could be attributed to the stable low velocity fluid film and vortexes between the crescent-like structures.

**Figure 8:**
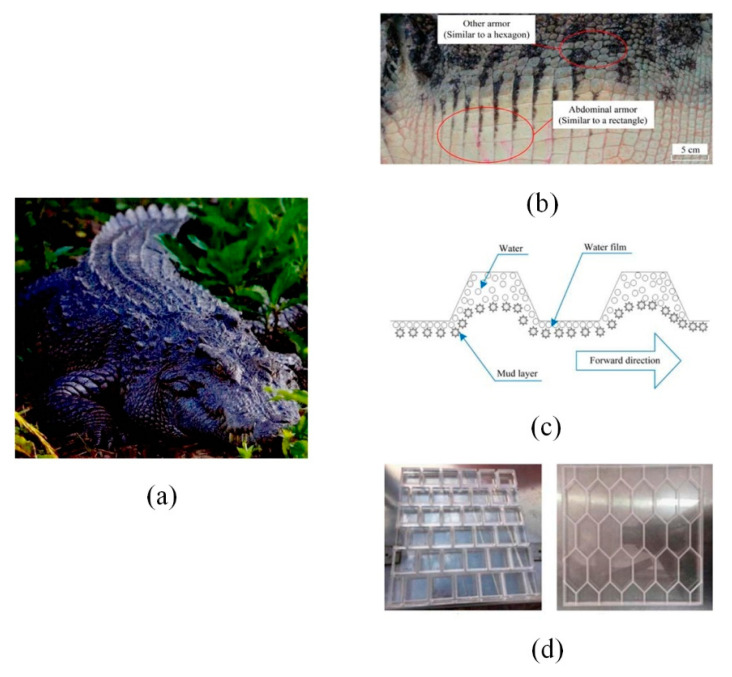
(**a**) The image of crocodile; (**b**) The crocodile’s armor structures [83]; (**c**) Drag reduction mechanism diagram based on water film theory [83]; (**d**) Two kinds of bionic ship board [83].

Most crocodiles live in swamps (Figure 8a). When they crawl in the swamp, the abdominal armor structure with macroscopic gully can introduce water and thicken the water film to reduce travel resistance (Figure 8b). This coincides with the water film theory for drag reduction which is applied to the design of ship-type paddy field machinery (Figure 8c). Yan et al. [83] imitated crocodile to design the rectangular and hexagonal ship boards (Figure 8d), and obtained drag reduction rate of 6.3% by experimental verification.

### 3.2. Concave-Convex Structure

**Figure 9:**
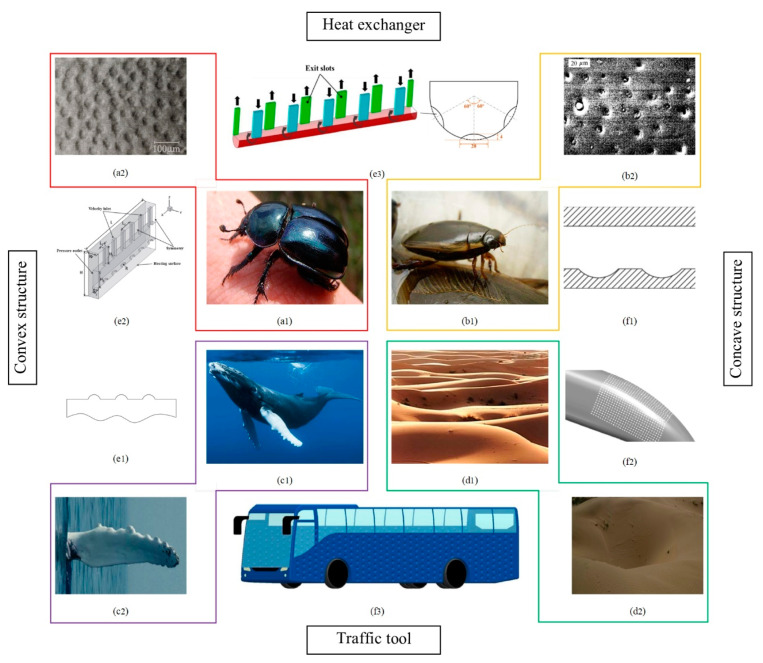
The image of the (**a1**) dung beetle, (**b1**) cybister bengalensis, (**c1**) humpback whale, (**d1**) dune topography; (**a2**) The SEM image of the dung beetle head [84], (**b2**) The SEM image of the cybister bengalensis back [85], (**c2**) The structure of humpback whale fins [86], (**d2**) The pits in the desert; (**e1**) The convex rough surface of the MIRA model, (**e2**) The microchannel heat sink model with impinging jets with convex dimples [97], (**e3**) The newly developed swirl chamber with protrusion structure [99]; (**f1**) The cross-section of microchannel with dimples [96], (**f2**) The non-smooth surface with ball sockets of train model [91], (**f3**) The commercial vehicle model with concave structures [94].

The dung beetle living in soil, cybister bengalensis and humpback whales living in water all have concave-convex structures on their body surfaces to reduce movement resistance [84,85,86], as shown in Figure 9(a1,b1,c1). Besides, Figure 9(d1) shows that wind-shaped dunes always take on a hemispherical appearance, allowing them to suffer as little drag as possible [87].

On the one hand, the secondary flow generated in the concave region contacts with the main fluid to result in vortex cushion effect which leads to the decreases of velocity gradient and shear stress near the wall. On the other hand, the low-velocity flow zone formed between the adjacent convex structures and the backflow appeared downstream of the convex structure can reduce the direct liquid-solid contact area, increase the thickness of the boundary layer and thus decrease the flow resistance [88,89,90].

Table 4 shows the research work of several concave-convex structures in recent years. Zhu et al. [91] conducted numerical simulation on the simplified CRH3 high-speed train model with ball sockets. It found that the aerodynamic drag of the train decreased at first and then increased with the increases of the radius, depth and array distance of ball sockets (Figure 9(f2)). Li et al. [92,93] pointed out that the resistance could be reduced by arranging spherical pits in the specific positions such as the front and rear of the train, bogies and inter-car connections. Palanivendhan et al. [94] used dimples to improve the air flow around a commercial vehicle body and the drag reduction could only be achieved by adding a certain number of small size dimples (Figure 9(f3)). Yang et al. [95] carried out wind tunnel tests on the notchback MIRA model with pits, convex structures and grooves arranged on the different locations of the car (Figure 9(e1)). When pitted structures were arranged on the rear of the notchback model, the drag reduction rate was largest. Xu et al. [96] applied the concave structures to the structural optimization of traditional smooth microchannels and the flow resistance decreased with the increase of concave structure depth or the decrease of concave structure spacing (Figure 9(f1)). Huang et al. [97] studied the influences of concave structures, convex structures and mixed structures on the flow characteristic of the microchannel heat sinks with impinging jets (Figure 9(e2)). The results showed that the application of convex structures could minimize the flow resistance. Jing et al. [98,99] successively optimized the channel structures applied in jet impingement and swirl cooling, and pointed out that it was better to arrange the protrusion on the side of the nozzle for drag reduction (Figure 9(e3)).

### 3.3. Superhydrophobic Micro-Nano Structure

**Figure 10:**
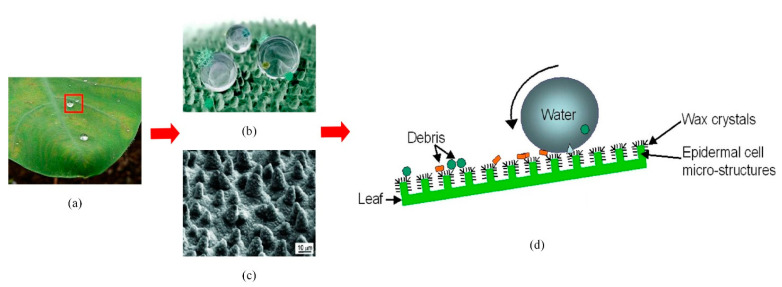
(**a**) The image of lotus leaf; (**b**) The diagram of amplified micro-structures; (**c**) The SEM image of lotus leaf [10]; (**d**) The diagram of droplets movement on lotus leaf.

The superhydrophobic surface has the advantage of flow drag reduction. The lotus leaf is a typical superhydrophobic surface (Figure 10a). It has a hierarchical micro-nano composite structure consisting of papillary epidermal cells and mirror-like waxy crystals [10,100] (Figure 10b,c). This hierarchical structure can absorb the air and make the gas-liquid contact replace part of the solid-liquid contact. When the fluid flows on the superhydrophobic surface, the slip motion occurs which reduces the velocity gradient and shear stress on the boundary surface, delaying the change of flow state near the laminar flow, and then decreasing the viscous resistance. As a result, droplets can easily slide off the lotus leaf and carry away contaminant on the surface [101] (Figure 10d).

Researchers have studied lots of superhydrophobic surfaces by imitating the superhydrophobic micro-nano structure of the lotus leaf [102,103,104,105]. Tuo et al. [106] established a superhydrophobic aluminum foil surface with the contact angle of 160° and achieved a drag reduction rate of 30%. Li et al. [107] manufactured a superhydrophobic aluminum surface with a drag reduction rate of 19.2%. Rajappan et al. [108] prepared aluminum substrates with different surface textures and sprayed a mixture of high molecular weight polymer binder and low surface energy hydrophobic agent on these aluminum substrates to obtain superhydrophobic surfaces. The maximum drag reduction rate of these surfaces was 26%. Through layer-by-layer coating using adhesive tape and the superhydrophobic paint made up of H, 1H, 2H, 2H-perfluorooctyltriethoxysilane (PFOTES), TiO_2_ nanoparticles and ethanol, a robust superhydrophobic surface with a drag reduction rate of 12.7% was fabricated by Hwang et al. [109]. Liu et al. [110] prepared a multilayer superhydrophobic organic-inorganic composite film based on the metathesis reactions of disulfide bonds and hot pressing of fluorinated silicon particles. The composite membrane exhibited excellent drag reduction property up to 27.7%.

## 4. Machining Methods for Biomimetic Structures

In general, the biomimetic structure is in the micro-nano scale. Compared with the macroscopic structures, the micro-nano structures usually present novel physical and chemical properties. In order to realize the research and application of micro-nano structure characteristics, it is necessary to strictly control the material growth and machining accuracy. Therefore, it is indispensable to summarize and develop the existing micro-nano processing technologies. There are many machining methods that can be used in the processing of biomimetic structures with the micro-nano size [111]. Furthermore, some methods can be used in combination to obtain biomimetic structures with higher accuracy. Four typical bionic micro-nano processing technologies are introduced.

### 4.1. Photolithography

**Figure 11:**
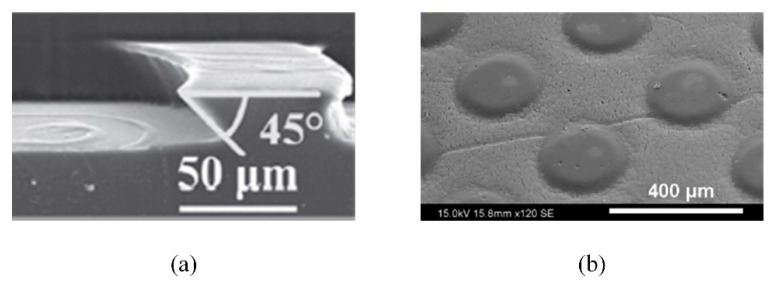
(**a**) The section of the inclined arc pitted groove [113]; (**b**) Morphology of the hydrophilic bump surface and hydrophobic background [114].

Photolithography is one of the most efficient methods for fabricating micro-nano structures. It mainly relies on the photochemical reaction between light and photosensitive substances, as well as the selective removal of materials by physical and chemical methods to produce complex structures [112]. Chen et al. [113] adopted two successive steps of UV lithography to fabricate the inclined arc pitted groove that imitated the curved outline and wedge-shaped holes of nepenthes alata (Figure 11a). Based on moisture collection of the Namib desert beetle, Moazzam et al. [114] constructed hydrophilic polydopamine bumps on hydrophobic polypropylene films through negative photolithography (Figure 11b). Photolithography has the advantage of being able to process micro-nano structures as small as tens of nanometers. The shape and size of the structure can be precisely controlled. It is easy to make photo masks which can be used repeatedly. However, it requires expensive equipment. The operation process is relatively complex and the processing materials are limited to some extent [115,116].

### 4.2. Nanoimprinting

**Figure 12:**
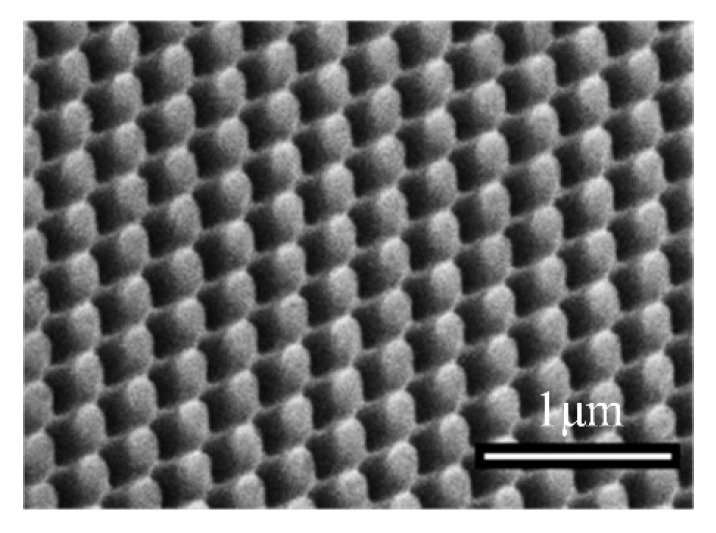
Micrographs of the PMMA surface [120].

Nanoimprinting is a graphic transfer technology, which applies the traditional mold replica technique to a micro-nano machining field directly. The realization of original graph transfer is to make the template with nano structure closely contact with the imprint resist coated on the substrate through external mechanical force. After demolding, the final graph transfer is realized by removing the residual imprint resist by etching [117,118]. By applying nanoimprinting, Saison et al. [119] prepared PDMS masks with microstructures inspired by the lotus leaf and butterfly wings, and then transferred the micro-nano structures onto MTEOS films on the surface of silicon or glass substrates. Dickson et al. [120] have imprinted the nano cylindrical array with different diameters and heights on the PMMA surfaces through imitating cicada wings (Figure 12). Nanoimprinting is not affected by the optical diffraction limit, and the highest resolution can be less than 5 nm. The simple technological process of nanoimprinting also provides the possibility for the large-scale fabrication of nanostructures. However, the preparation process of the mask is relatively complex and deformation can easily occur in the processing procedure [121,122].

### 4.3. Femtosecond Laser Processing

**Figure 13:**
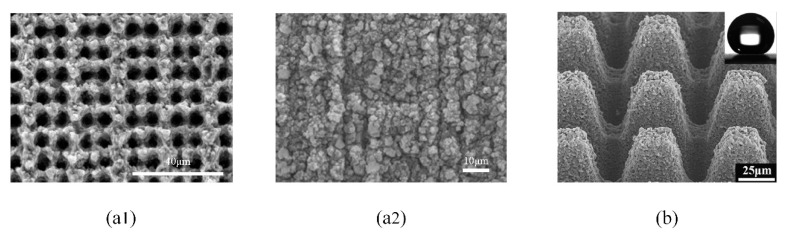
The SEM image of (**a1**) the rough silicon surface [127], (**a2**) the rough PDMS surface [127]; (**b**) the original SMP micropillar array [128].

Femtosecond laser processing is widely concerned in the field of micro-nano structure preparation because it highly conforms to the environment-friendly and resource-conserving concept of green manufacturing [123,124]. The energy of the pulse laser is absorbed when the femtosecond laser acts on the surface of the material. Then, the bound electrons become high temperature free electrons and accumulate rapidly following the nonlinear ionization mechanism. The material in the laser action region is stripped off the base metal surface in the form of plasma jet after free electrons reaching a certain density [125,126]. Yong et al. [127] fabricated the micro-nano hierarchical rough structures inspired by fish scales and lotus leaves respectively on the surfaces of silicon and PDMS by femtosecond laser processing. A superhydrophilic periodic hierarchical micro-mountains array was formed on the silicon surface (Figure 13(a1)). The surface of PDMS was covered with a high number of superhydrophobic bumps decorated with masses of nanoscale protrusions (Figure 13(a2)). Bai et al. [128] modified the hierarchical micropillar array on the SMP surface with fluoroalkylsilane (Figure 13b). Moreover, the superhydrophobicity of SMP surface could be reversibly weakened and recovered due to the macro/microscopic shape-memory effect in response to heat. On the one hand, femtosecond laser processing has the characteristics of simple machining processes and non-contact. On the other hand, it has ultra-short pulse width and ultra-high peak power. Therefore, its processing accuracy can reach hundreds of nanometers. It can process complex structures with a wide range of solid materials and the machined sample is difficult to deform. However, Femtosecond laser processing is working with many interacting parameters, which should be repeatedly debugged and reduces the processing efficiency [129,130].

### 4.4. 3D Printing

**Figure 14:**
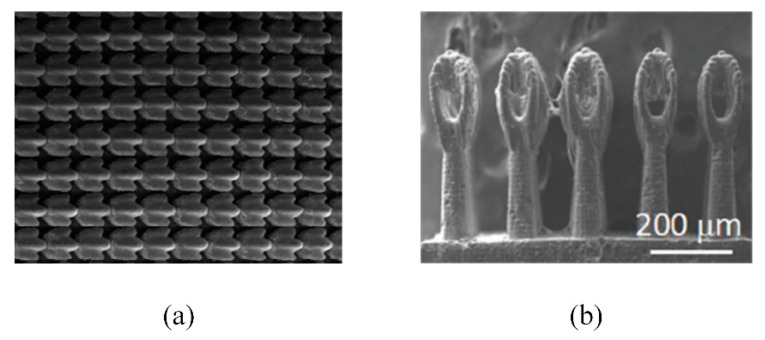
(**a**) The SEM image of the fabricated synthetic shark skin membranes [134]; (**b**) The SEM image of 3D-printed eggbeater hair structure [135].

3D printing is a rapid prototyping technology which stacks materials from point to line, line to plane and plane to volume discretely. It obtains a 3D model through computer-aided design software construction or entity scanning. After optimizing the structure and printing parameters of the 3D model, it is transmitted to the printing equipment for additive manufacturing. Finally, the printed product is finished through post-processing [131,132,133]. Wen et al. [134] constructed the shark skin denticles imitated the shortfin mako on the flexible membranes by 3D printing, and conducted hydrodynamic tests on it (Figure 14a). Inspired by salvinia molesta leaf, Yang et al. [135] printed superhydrophobic micro-scale artificial hairs with eggbeater heads. 3D printing has high processing accuracy up to tens of nanometers (Figure 14b). It can quickly manufacture products with complex structures and fabricate whole products, but still not be mass produced due to time and cost limitation [136]. However, the processing quality is affected by size deviation and parameter selection, which cannot be observed and characterized in real-time. There are lots of materials available for 3D printing, but the materials that can be processed by specific printing equipment are limited. It is difficult to process a variety of composite material structures at micro and nano scales simultaneously [137].

## 5. Summary and Outlook

In this paper, six biomimetic structures in heat transfer and fluid flow fields were summarized. A series of structural factors were discussed, such as the shape, arrangement, size and so on. Four typical micro-nano machining technologies were introduced. In general, the biomimetic structures could improve the heat transfer and/or flow performance more or less. The biomimetic structures could be manufactured by the present micro-nano machining technologies. The prospect of biomimetic structure optimization was proposed as follows:

(1) It is necessary to consider the synergistic effect of multiple structures on the biological surface. For example, the fish control the external flow field using the streamlined body, scales and flexible fins. It is partial to analyze one of the fish structures. In addition, combining the biomimetic structures with the existing traditional enhanced heat transfer structures is also worthy of further researches.
(2) It seems that the simplified biomimetic structures show good performance as well as actual biomimetic structures. The heat transfer enhancement and drag reduction mechanism is a guide for simplifying biomimetic structures reasonably.
(3) The structural parameters of the biomimetic structure are the most important factors. We are a long way from establishing the functional relationship between the structural parameters and heat transfer coefficient or flow resistance of biomimetic structures for performance evaluation.
(4) The surface force is the dominant instead of volume force on fluid flow for micro heat sinks. The biomimetic structures are applied in microchannel, which is a major challenge to research micro heat transfer.
(5) The composite biomimetic micro-nano structure is the main developing trend. It requires higher precision and quality of micro-nano machining technology. It is worth exploring the present machining methods used in combinations.

## Figures and Tables

**Table 1:** Summary of research work for fractal heat exchangers.

Reference	Research Method	Dimension	Medium	Factor	Maximum Heat Transfer Enhancement Rate	Application
[19]	simulation	microscale	deionized water	channel aspect ratio (*α* = 0.3–1)	20%	microchannel heat sink
[20]	simulation experiment	microscale	deionized water	structure layer (0–5)	110%	microchannel heat sink
[21]	simulation	microscale	deionized water	structure shape (smooth; ribbed; concave)	17%	microchannel heat sink
[22]	simulation experiment	conventional scale	deionized water	structure shape (Y-type; H-type; conventional spiral)	23%	spiral-tube heat exchanger
[23]	simulation	conventional scale	deionized water	structure shape (lung patterned; corrugated)	71.3%	plate heat exchanger

**Table 2:** Several hybrid wetting structures.

Reference	Research Method	Hybrid Wetting Structure	Contact Angle	Factor	Maximum Heat Transfer Enhancement Rate
[56]	experiment	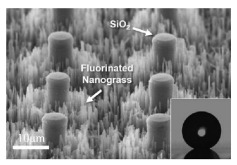	hydrophilic: 24.6° hydrophobic: 167.1°	wettability (hydrophilic; hydrophobic; hybrid wetting)	63%
[57]	experiment	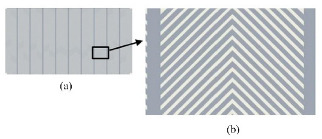	hydrophilic: 73° hydrophobic: 125°	wettability (hydrophilic; hydrophobic; superhydrophilic; hybrid wetting)	9%
[58,59]	experiment	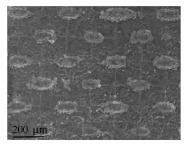	hydrophilic: 22.6° hydrophobic: 135°	wettability (hydrophilic; hydrophobic; hybrid wetting)	107.3%
[60]	experiment	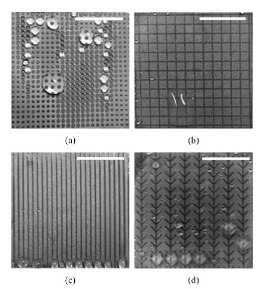	hydrophilic: 0° hydrophobic: 161°	surface inclined angle (0°–90°) superhydrophilic pattern shape (dot; mesh; line; branch)	50%
[61]	experiment	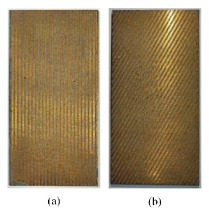	hydrophilic: 86.4° hydrophobic: 151.2°	hydrophilic parallel-stripes pattern inclined angle (60°; 90°)	114%
[62]	experiment	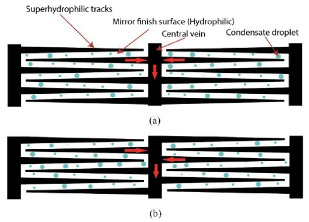	hydrophilic: 78.2° hydrophobic: 161.2°	superhydrophilic tracks fractional area (0–50%) superhydrophilic tracks spatial layout (interdigitated; staggered)	35.9%
[63]	experiment	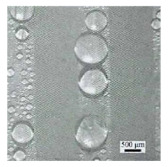	hydrophilic: 0° hydrophobic: 158°	superhydrophilic region width (0.8 mm; 1.33 mm; 2.07 mm)	39%

**Table 3:** Several biomimetic shark-skin grooves.

Reference	Research Method	Groove	Medium	Factor	Maximum Drag Reduction Rate
[73]	simulation	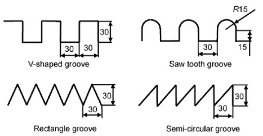	deionized water	groove shape (V-shaped; saw tooth; rectangular; semi-circular)	30%
[74]	simulation	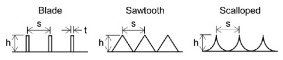	deionized water	groove shape (blade; sawtooth; scalloped) dimensionless spacing (*s^+^* = 0–50) dimensionless height (*h^+^* = 0–15)	13%
[75]	experiment	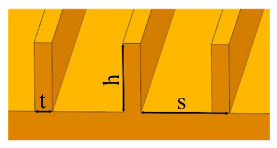	air	dimensionless spacing (*s^+^* = 7–35)	6%
[76]	simulation	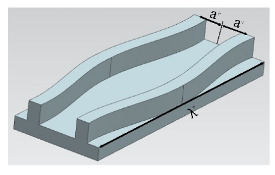	air	dimensionless amplitude (*a*^+^ = 0–18.47) dimensionless wavelength (*λ^+^* = 107.9–431.6)	9.8%
[77]	experiment	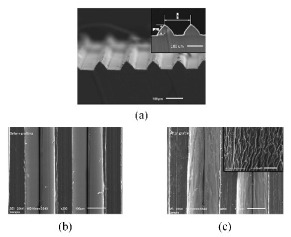	deionized water	drag reduction agent (polyacrylamide)	14%

**Table 4:** Summary of research work for concave-convex structures.

Reference	Research Method	Medium	Factor	Maximum Drag Reduction Rate	Application
[91]	simulation	air	ball socket radius (20–180 mm) ball socket depth (4–16 mm) ball socket array distance (100–500 mm)	25.19%	high-speed train
[94]	simulation	air	dimple size and number	4%	commercial vehicle
[95]	experiment	air	structure location (top; luggage hatch; rear; bottom) structure shape (pitted; convex; grooved)	2.26%	notchback
[96]	simulation	deionized water	concave structure depth (*d* = 0.05–0.2 mm) concave structure spacing (*s* = 0.7–2.8 mm)	2%	microchannel heat sink
[97]	simulation	deionized water	structure shape (convex; concave; mixed)	9%	microchannel heat sink
[98,99]	simulation	deionized water	structure location (nozzle side; slot side)	5%	swirl chamber

## Symbol Description

*α*	Fractal channel aspect ratio
*p*	Interspace of conical column structure
*d*	Tip size of conical column structure
*h*	Height of conical column structure
*D_w_*	Departure diameter of droplet
*ρ_w_*	Density of droplet
*s^+^*	Dimensionless spacing of groove
*h^+^*	Dimensionless height of groove
*a^+^*	Dimensionless amplitude of sinusoidal groove
*λ^+^*	Dimensionless wavelength of sinusoidal groove
*d_c_*	Depth of concave structure
*s*	Spacing of concave structure
CAD	Computer aided design
SEM	Scanning electron microscope
UV	Ultraviolet
PDMS	Polydimethylsiloxane
MTEOS	Methyltriethoxysilane
PMMA	Polymethyl methacrylate
SMP	Shape-memory polymer
